# Supplementary material for: Sosuga Virus Detected in Egyptian Rousette Bats (Rousettus aegyptiacus) in Sierra Leone
Source: Viruses. 2024 Apr 22;16(4):648. doi: 10.3390/v16040648 (PMC11054331; doi:10.3390/v16040648)
Supplement: Supplementary file 1 [file viruses-16-00648-s001.zip › Table S1.pdf]

Table S1. Sosuga virus-specific enrichment oligonucleotides. Enrichment oligos, 80 base pair oligonucleotides, used to generated Sosuga virus (SOSV) sequences using in-house scripts (GitHub - evk3/Nipah_phylogenetics: Collection of scripts used for "Inference of Nipah virus Evolution, 1999-2015").

**Oligo Name (sequence-name_start_stop)**	**Oligo Sequence**
NC_025343_Sosuga_2012_M_0_80	ACCAGAGGGAAAATTCAGAAGGGTCGTTCTCGATAGAGGAAGGTCCTGATATTGTGAGCCCGGAAGTGGCAAGGCTCGGA
NC_025343_Sosuga_2012_M_81_161	TCGAAATCGACTTTGAGCTTGTTCTTCCGGAGCCTCTGACTTGCCTGATTTCATAAGTCAGCCTTTCTCTGTTGTTGGAA
NC_025343_Sosuga_2012_M_162_242	CCATGGCGTCCATATTCAGAGCACTTGAACAATTCACTCTTGATCAGGATGGAGGGGGGAGAGGGGGATTCGCTGACCAG
NC_025343_Sosuga_2012_M_243_323	CGCCTGAGACATTGCAGGCAACTATCAAAGTCTTTGTCATCAATACTCCTGATCCAAATCTTCGCTATAGAATGATGTGC
NC_025343_Sosuga_2012_M_324_404	TTTGCTTGCGCTTAGTAGTTAGTAATTCGGCAAGAACTGGGCAGAGACATGGAGCACTCATGACACTGCTTAGCTTGCCC
NC_025343_Sosuga_2012_M_405_485	CTGCAGTGATGCAGAATCATTTTAGGACTGCTGAACGCTCACCTGACTGCCAAATCGAAAGAATTGAGGTCGATGGATTC
NC_025343_Sosuga_2012_M_486_566	AACCGGGAACATACCGCATCAGGCCCAATGCAAGAACACCGCTGACAGTAGGGGAGGTTGTTGCTCTTGAAGAAATGGCA
NC_025343_Sosuga_2012_M_567_647	ATGATATCCCAGAGGCATTGGCTAATAACACCCCTTTTATTGATGCCAACACCGAGATGGAAGACTGTGATGAAACTGAG
NC_025343_Sosuga_2012_M_648_728	AATTCTTAGAGGCAATTTACAGCACCCTTATCCAAGTTTGGATCATGGTGTGCAAAAGCATGACCAACTTTGACCAGCCT
NC_025343_Sosuga_2012_M_729_809	CCGGGTCAGACGAACGCAGAGTTGCAAAATACCAGCAACAAGGTAGGCTGAACCAGCATTACCTGTTGCAGGGAGAGGTA
NC_025343_Sosuga_2012_M_810_890	GGCGAAGAATACAGGTAGCAATAAGAGAGAGTCTTCCAATCAGACAATTTTTAGTCAATGAGCTCCAGATTGCAAGCAAC
NC_025343_Sosuga_2012_M_891_971	AGGGACCCATTACAGGCAAGTACTATGCTATGGTTGCAGACATTGGTAAATATATTATGAATGCAGGAATGGGAGGCTTC
NC_025343_Sosuga_2012_M_972_1052	TCATGACAATTAGGTTTGCTTTGGGACAAAAATGGCCACCGTTAGCTCTTGCTGCATTCTCAGGTGAGATTGTCAAGATT
NC_025343_Sosuga_2012_M_1053_1133	AAAGTCTCATGTTACAGTATAGAAGGTTGGGTGAGAGGGCCAAGTATATGGCTCTTTTGGAGATGTCAGAAATGATGGAT
NC_025343_Sosuga_2012_M_1134_1214	TTGCAGCAGCAAATTTCCCACTCCTATACAGTTATGCAATGGGAATCGGAAGTGTCCAAGATCCTCAAATGCGAGGCTAT
NC_025343_Sosuga_2012_M_1215_1295	CATTTGGCAGACCTTACTTGAATGCTGCATTTTACCAATTGGGGGTTGAGACTGCCAATCAGCAGCAAGGCTCAGTGGAC
NC_025343_Sosuga_2012_M_1296_1376	AGGAGATGGCAGAAGAGTTGGGACTCACCGAAGCAGACAAGCGGGCAATGGCGGCCACTGTTGCACGACTCACAACAGGA
NC_025343_Sosuga_2012_M_1377_1457	GAGGACAACAAGGAGGCAACATTGGGGTCAATGCAATGGCTCGGAGAGGGCAAGCCCAACCGCCCAGACAGCAAGCAATT
NC_025343_Sosuga_2012_M_1458_1538	ATAATCTAATTGATGAAGAGGAAGAGGATGAAGAAGAAGAGGAAGAAGAGCAGAATCTTGATGCAGAAATAGAAAGA
NC_025343_Sosuga_2012_M_1539_1619	GATTTGCACTCAGGTTAGCACAGGAGCAGGAAAGATGGGGACGCCGCCTGGCCGAACTCGAAGCCGAAAGGGCACAGACA
NC_025343_Sosuga_2012_M_1620_1700	GACAAGGGCCAGGGGCAGCACCCCAGTCTGGGCAACACCAAAACCAGACAGACAATGACCAATCAGAATACAACAGCAAT
NC_025343_Sosuga_2012_M_1701_1781	TCGCAGAATAGCAAGGCTTTCAAACAACGAGCGGGCTTGCAGCTTATACGCATTTTCTTGCAACTTTACAGTTATAGGCT
NC_025343_Sosuga_2012_M_1782_1862	TTAAGAAAAAACTACTATAGGCCCGAAGGTTGGCAACTTTCGGGCCCCCCTCAGCCTTGGCTCCAAGGCGCCCCCCCAAT
NC_025343_Sosuga_2012_M_1863_1943	CACCCACAAGCCAGCACTAACAGCCACAGCACAACTGACAGAAAATGGACCAGCCACCATCAGATGCGGAAATTTCCGCC

NC_025343_Sosuga_2012_M_1944_2024	GGATAGAGAGGGGACTGGCCACTGCCAGGCACTTCGCACCAGGCCCAGTCACGTCCCAAAGCAGTCTTGGGAAGTCCACC
NC_025343_Sosuga_2012_M_2025_2105	TCAAGAAGGGAAACACAAAACAATTGGTTGACTCCGCGGAATTCACCGCCGCAACACTGGCGCCCGCAGGAGGACTGCAG
NC_025343_Sosuga_2012_M_2106_2186	GATCAATGCCCTGCTCGATTCCAGCAGGGCAATCTAAGGCAGGGCAAGGCGCCCGGCCAAAGGTTAAGAAAGCTGCCCAA
NC_025343_Sosuga_2012_M_2187_2267	CTCGAGCGACGCCCACTAAACCTGATGCTCCTACAATTGAACCAGTGTATGAGGACATAGTATCCACTCCTGGTAGAGAC
NC_025343_Sosuga_2012_M_2268_2348	AAGCACAAGTGCAGCTTCCATCCCCAAAATTGAGCGCAAAAGACAAACTCCTATCGGCCCCGCCACTCCAGCAACATTCC
NC_025343_Sosuga_2012_M_2349_2429	ATATCCCCACTGGTCCGGAGGGACAAAACTTTAAGAGGGGGCTGTCCTCAGGAAGCGCCGCCTCTATTAGAAGAAACCCC
NC_025343_Sosuga_2012_M_2430_2510	ATCCAGGACACAGACGAGAGTTCGATCTTGTATGGGGAGGAGGATCATTCACAATGCGAGAGTGGTGCAATCCAACCTGC
NC_025343_Sosuga_2012_M_2511_2591	GTCCAATCACACCAATCCCCAGGAGACATCACTGCAGATGTGGTCAGTGCCCTCGAGTCTGCAAACAGTGTGAAGGAGAT
NC_025343_Sosuga_2012_M_2592_2672	ATCAGGTACTTGAAGGTGATGGAGAACAAGATGAACCAGCTGGAGTGGAAAATTGACAAAGTATTGGCTCAAAACAATAT
NC_025343_Sosuga_2012_M_2673_2753	ATACAGCAGATTAGGAACGAACAAATGGTTCTCAAAGCAGGGATGGCTACACTTGAAGGGCTGATAACCACGATAAAAAT
NC_025343_Sosuga_2012_M_2754_2834	ATGGATCCCGGAGTCGGCACGGGAGCTGACGCTGCTCAAGCAAAGCGTGCATTTAAGGATGTGCCAGTAGTAGTCAGCGG
NC_025343_Sosuga_2012_M_2835_2915	CCTGTTATTGGGGAGAATGATCTCATCTTTGAGTCCAAACTAGAAGTCGGTAATCTAGGAAGGCCTCAAAAGGTCAATCC
NC_025343_Sosuga_2012_M_2916_2996	ACACCCCGACGCCGTGGAGTCCCGACCGAATCCGAATTGGCAAGTTACAAATTGACTCTAGCAAAGTTACTCAAAGACTG
NC_025343_Sosuga_2012_M_2997_3077	ATCCCTAATAACACAGCACAATCAAAGTTCCTTGACGCGATTGAAAAGATAAAAACTGAGGCTGACTTTAAGGCGCTCAA
NC_025343_Sosuga_2012_M_3078_3158	CGAGAGATAGTACGTGCTGCAGTGTGAAAACTGTCATTGACTATCGCTTCATATCTCAACACTTTTTGCAATCTTAGCCT
NC_025343_Sosuga_2012_M_3159_3239	ACTGGTTTCTCTGCTTGCACTATCCGCACAACATCTCAAAACAAAAAGCATTCACCTCTGTTATTGCAAATGCCAACCAC
NC_025343_Sosuga_2012_M_3240_3320	AAAAAAATCATAACCTGAAGGTCTTTAAGAAAAAACTAAGGTGGGTCCGAACCCGTTCGACCCCGACCGACCCCAAAATG
NC_025343_Sosuga_2012_M_3321_3401	CGCACCGCCAAGCAACGATTCCTGTCCATGTGGATCACCACAGTGAGAAGAATCATCTCCGTGCTTTCCCGATTGTCCAA
NC_025343_Sosuga_2012_M_3402_3482	CGGATTCACCTGAGGGCACAGAAAAGGGGCGGTTAATTAAACAGATGAGATTCAAGGATCTAACTCCTAGGGGCTCAACT
NC_025343_Sosuga_2012_M_3483_3563	AACCCCCTATATCATTCATAAACACCTACGGGTTCATAAAACCACTTAGGACCAGAGAGGAATTCTTCTCAGAGTTGCAT
NC_025343_Sosuga_2012_M_3564_3644	AGCAATCACAGACCCCCTGTCTCACTGCATGCAGCATACCATTCGGAGCAGGCCCAGCGCTGGATCATCCGGAAAGACTC
NC_025343_Sosuga_2012_M_3645_3725	TGGACGAAATTGAGAAGGCTTTAATAGTTGTGAGGAAGAGTGCCAGCACAAGTGAGGAGTGTGTATTTGAAATCAGGAAA
NC_025343_Sosuga_2012_M_3726_3806	TTCCCCCGATGCTGACCAGGCACCAGCTTGCGGGGAACAAGATCCTATGTGTTCCATCTGATAAATATGTTAAGGCTCCT
NC_025343_Sosuga_2012_M_3807_3887	GGAAATTGACCTCAGGTGTGGATTACGCTTACCACATTGTCTTTATATCTGTCACACTGTGCCCTCCTAGTCAAAAATTC
NC_025343_Sosuga_2012_M_3888_3968	GGGTACCTATGCCGGTCCAAAGCTTGCGTGCAAAAGTCATGAGGAGTGTGCATTTGGAAGTCATGTTAAAGATTGACTGT
NC_025343_Sosuga_2012_M_3969_4049	ATCCGGGTTCCCCTATAACCAAAAATTTGATATATGATTCTGAGAATGATGTTTGGCTTGCTTCCATCTGGTTCCATCTA
NC_025343_Sosuga_2012_M_4050_4130	GTAATCTATACAAAGGCCATAAGCCCTTCAAAGAATACGATGACCATCATTTTGCTGCAAAGTGCAGGTCAATGAAACTG
NC_025343_Sosuga_2012_M_4131_4211	AAGTAGGGATTGTTGATCTATGGGGTCCCACGTTTCTTGTTAAGTCCCACGGGAAAATACCCCATGCAGCTCGGCCTTTC
NC_025343_Sosuga_2012_M_4212_4292	TCGGAAAGCATGGTTGGGTCTGTCATCCCATAATGGATTGTGCACCTGCAATTTGCAAGTCATTGTGGGCACTGAGTGTA
NC_025343_Sosuga_2012_M_4293_4373	CTATTGTGCAAGTAACTGCAGTCCTACAGGCATCAGATTTGTCTCAAATGGTCCGAATGACAGATGTGATTTTCCCGAAA

NC_025343_Sosuga_2012_M_4374_4454	TAAAAGTTAATCCGGACCTACACGGCCTTGGCAAATCCAGATGGAACCCAGTAACCAAAATAGTCAGCCCCGAATAAAGA
NC_025343_Sosuga_2012_M_4455_4535	ACCACAACTAGGTGCAACAAAAGCATCAAGAACCTGAGACATCCCCCAACATTCCAAAGACAGAGTCCAAACCTCGGCAT
NC_025343_Sosuga_2012_M_4536_4616	TGACCCATAATCAATATTGCTGCAAGACTGCATAGAAATGCTTCTGCTCTAAGCTATAGCGGTAGCCTGCAAGTTAATAG
NC_025343_Sosuga_2012_M_4617_4697	AAGAGCTCTCACATCACTATCTGCATTATCAAATCTGCACACTCATTTCTCAACGCTTGCTTTACCAATCTTCAGATTTA
NC_025343_Sosuga_2012_M_4698_4778	GAAAAAACCTAATCTCAATTACAAATGCAGAATTGAGCAGTCTAAATTGGGTCCGAACCCGTTCGGACCCGAACCCGAGA
NC_025343_Sosuga_2012_M_4779_4859	ACCAAGCGAAGACATCCACTCCCCCCCCCAACCCCAAAGGAAGCCGACTCCAAAAAGAGGACTCCACACAATGGCACACA
NC_025343_Sosuga_2012_M_4860_4940	AAACTCATTAATTTTACTTCTAATGTTGACAGAGACAGGGAGTTCTGTGAATATTCAGCTGCTTCAGTCACTGGGAGTAA
NC_025343_Sosuga_2012_M_4941_5021	CAACACTCATAAACGCCAACTTGCATTTTATAATCAACAACCTCCGTCATACCTCGTAATTAGACTTCTGCCGACACTAA
NC_025343_Sosuga_2012_M_5022_5102	TATTCCTAAGCAGAATTGCACTCTTAATAGTATTAACAGGTATGAAAAGGCTGTCAAAGATATAATAAAACCCATTAGTG
NC_025343_Sosuga_2012_M_5103_5183	CAATCTCAATTGGCTTTCTGACAATTTAATTCCAAAAAGAAGAGGGAAAAGATTTGCGGGGGCAGCTATAGGACTTGCAG
NC_025343_Sosuga_2012_M_5184_5264	ACTCGGTGTTGCAGTTGCTGCACAGGCAACAGCTGCAGTAGCTCTAGTGGAAGCCAGAGCAAATGCAGAGAAGATTTCCT
NC_025343_Sosuga_2012_M_5265_5345	AATGTCAGCAGCCCTACAAGAGACGAACCAAGCTGTCTCTTCTTTGACAGCTGCCATGGCGAGTAGTGGTATTGCAATTC
NC_025343_Sosuga_2012_M_5346_5426	AGCAATACAGAATGAGATCAATAATGTGATTCATCCTATTCTCAACCAGGTACAGTGTGGGGTACTAGACTCACAAATTG
NC_025343_Sosuga_2012_M_5427_5507	ATCCATCTTAAACTTATATCTCATAAAGATCACAACCATCTTTAACAATCAACTAACAAATCCTGCTCTCCATAGAATAT
NC_025343_Sosuga_2012_M_5508_5588	GATCCAGGCATTGTCTGTTCTAATGCAAAGTACTAAAGACTCACTCAAAAACCTCACAGCCGGAGATACCCAAACCTCAC
NC_025343_Sosuga_2012_M_5589_5669	GGATCTGATAAGAACCAATCTAATAGAGGGCCAGATTGTTGCAGTCAACATGACAACGCTTCAGATGGTTATAGCAGTGT
NC_025343_Sosuga_2012_M_5670_5750	CATCCCGGCGGTTGCGAAGTTGGAGTCAGCTGTCCTCTTAGATTTTATTTCAATTACTGTCAGCTCTAATCAATCTGAGG
NC_025343_Sosuga_2012_M_5751_5831	AATGCTGCAGCTGCCCTCAAGAATATTAGAGGTCGGAAATAACATATATACGTTTAAGGGAGATCAGTGCACATTAACTG
NC_025343_Sosuga_2012_M_5832_5912	AACTACAGCTTACTGCTTATACAGTGATGCTGTCCCTGTCAATGAGAAAATTTCTGATTGCATGAAGGGAATTCAAAGCT
NC_025343_Sosuga_2012_M_5913_5993	CTGTATATTTACAAGGATCATAGGCTCCTTTGCTAATAGATTTGCCAGTGTGAATGGGGCAATATTTGCAAATTGTAAGA
NC_025343_Sosuga_2012_M_5994_6074	TTTAACATGTTCTTGTACCCAACCTGATGGGTTAATATATCAGCCCGATAATGTACCCTTGACAATCATTGACAAAATTA
NC_025343_Sosuga_2012_M_6075_6155	ATGTTCAAAGTTGAATATAGGACACCTGACCTTTAATATAAGAGATAGCACAAATGCTACTATTGATCTACATACTGATC
NC_025343_Sosuga_2012_M_6156_6236	GTCCGATTCTCAGATTACTATTACAAATCCTCTTGACTTATCCGCAGAGCTTACTCAAATTAATAATTCGGTAATTAATT
NC_025343_Sosuga_2012_M_6237_6317	CCACCTGCATCTTATGAATAGTGAAAATATATTGAGAAGAATTGATAGTGGACTATTCAGCAAGGCGATAATGATCTTCT
NC_025343_Sosuga_2012_M_6318_6398	GCTTGTGGGGGTCTGCTCTTTAATTATAGTTGTTATAGGTCTTATAGTCTGGATTAGGTTTATACTCCTCCATTTAAAGA
NC_025343_Sosuga_2012_M_6399_6479	TTCTTCACACACTAATCGCCAAGGAAGCAGTTTATTTTCTATTGATAGTGTATAACAATAGCAGTAGATAGAACATTATC
NC_025343_Sosuga_2012_M_6480_6560	ACTACACAGGTTGCCTAATGCATAATAGGTCTTTCAACCACTACTCTCCTATAAGTTAAAATCTGTTTTAATAAAAAACT
NC_025343_Sosuga_2012_M_6561_6641	ACAGTAAAAGTCAGGTAACTTGGGCTCGACCCAGTCAGCCCACCAGCCCACAAATCCCGAACGGACAGGGTCTGCCCTCA
NC_025343_Sosuga_2012_M_6642_6722	CAAGAACACCATGCATGCCAGAAACTCATCAGTCAGTAGCATCAGTGATTCCATCGACAATGTGTTTGGGAAAAGGAACA
NC_025343_Sosuga_2012_M_6723_6803	TCCTGTAATCAAAAGGACAGGAAAAAAATTATTTAGACTAGGCTCACTCATATTTCTAATAGTAATAATTAGCCTAACAG

NC_025343_Sosuga_2012_M_6804_6884	TAAGATAATAACTGAACTCTCTCTAGTTAAGAGTGAGTGTAGCAATAGAGACCATGTTACCGAAATAATCAATTTGCAGC
NC_025343_Sosuga_2012_M_6885_6965	AAAAGAACTCAGTCTGATGAATAACATCATAACAACCTTGAACACATTATTAACAACAACCACGGTTGACCTCCCGATTA
NC_025343_Sosuga_2012_M_6966_7046	ATTGACTAACTTTGGGAAAAGTATAGTTGATCAGGTGACAATGATGGTTAGACAGTGCAATGCAGTGTGTAGGGGCCCTG
NC_025343_Sosuga_2012_M_7047_7127	TGATAAACCAACCCAGAATATCCAATTATTCAACGGGCGCTACGCTATAATAAATAATTCAACAGCATATCCATCAAGAA
NC_025343_Sosuga_2012_M_7128_7208	TAGTATATCTGAACTTAAAGTACCAAGAGACTTTGTGCCTTCACCAGGCACCTTCCATGGGTGTTCAAGATTCCCGTCGT
NC_025343_Sosuga_2012_M_7209_7289	TAGTAACCATTATGGGTTATGGTGTTATAGTCATACTGTATCAAATGATACTTGTGACGGGTCTAATCCGTCTGTCCAAA
NC_025343_Sosuga_2012_M_7290_7370	CTTGTCTGTAGGGAAATTAATTACAGGGGATAATGGACAACCTGAACACAAGACTCTTTACACTCAGCAACTAAGCCAAA
NC_025343_Sosuga_2012_M_7371_7451	AGATCGGTTGTATCATTGCTCAGTGACTATGACCACTTTAGGTTGTTACATTTTATGCTCAAAGCCAAGGGTTAATGAGA
NC_025343_Sosuga_2012_M_7452_7532	CCAGGATTACGAGACAATAGGGATAGAGCCCATGATAATAGGTATGTTAGGGCTTGACGGAGTATATACTGATCTGGGTA
NC_025343_Sosuga_2012_M_7533_7613	CCCTGTAGGAATCTCTGACAATAGCTTATACGCAATGTATCCAGGCCCCGGGGGAGGTGTGATGTATAAGGATTTTTTGG
NC_025343_Sosuga_2012_M_7614_7694	CTTTCCATTGCATGGAGGGGTTAGATTCTCTGAGGCTTCCAAAATGTTAGGGAAGAACATCACTTTTCGGGGCTTCCCTC
NC_025343_Sosuga_2012_M_7695_7775	TAGTGACACATGCACTGAGCATGAAAAGAGCCTGACCCAAGAGCCGGCTAATATGCTAACAAGTCCTTATTACGGTGAGG
NC_025343_Sosuga_2012_M_7776_7856	TTTAGTTCTTGACTTTCTTTATGTGTGTACGTTATTGGATAACATACCTGGGGAGTGTAGTATACAGTTAATTCCTCCTG
NC_025343_Sosuga_2012_M_7857_7937	CAACATGACAATGGGCTCTGAATCAAAATTGTACAAATTGAATAACTCATTGCTACTTTATAAACGAAGCTCATCTTGGT
NC_025343_Sosuga_2012_M_7938_8018	GCCTTATACAGAAGTATACCAACTAAGTCTAAGGGTGTCCAAAAATAGCATGAAAGTGCGTGAATCAGTAAGGCTGAATA
NC_025343_Sosuga_2012_M_8019_8099	CACTTCTACTACTAGACCTGGAGTAGAAGGATGCAACATCAACAAAGTATGTCCTAAAGTATGTGTAACTGGAGTGTTTC
NC_025343_Sosuga_2012_M_8100_8180	GGCACCAGGAATTATAAGGAAAGCATTGAGTCCAAAGGAGTCTAATGAGGATCTGTTGTTTTTCCAGGCCTGGACTTCAG
NC_025343_Sosuga_2012_M_8181_8261	TTCTATAGCACGCCAAGGTCCCCTTATATCTCTATGTAGGGCTGATTCATGTGTACTGACCATCCCGCTAGGCAATTCTG
NC_025343_Sosuga_2012_M_8262_8342	TGTTTTCATTGGATACACAGATTCATTCTGTCTATCAGATAGAGATAATGAGAAGATCTACTGTGTTGCATTATTAGAAC
NC_025343_Sosuga_2012_M_8343_8423	AGATAACATGCCTTACTCTGAGATGACAATCAGATCTTTTTTGTACCTTATCAAATAACAATGTAAGAAAGAGAGTCACT
NC_025343_Sosuga_2012_M_8424_8504	GAACATACAACATGCTAGGAGCTCAGGTTACATGGTAATTATCTAGAGTAGGATGCAAAGCAATTTTAATCTTCTTGATG
NC_025343_Sosuga_2012_M_8505_8585	CTCACTCATAATATTAACTTCTTCGACTCAACTATCCCCCAAGAAAAAGCTACGCTATGCTTAAATTCTTCTTTAAGAAA
NC_025343_Sosuga_2012_M_8586_8666	AAGATGGGCCAGAATGGCTGCCCCGGCCCAGATCATCCTCCCTGAGGTGCATCTGGACTCTCCCATTGTTGAGAACAAGT
NC_025343_Sosuga_2012_M_8667_8747	GCTATACCTAATCCAACTGGGAGAATTACCAACTTATGATTATTACTACAATCATGACTATTTCCCAGGCATTGATTGGA
NC_025343_Sosuga_2012_M_8748_8828	CCGAATCAGGCATGAAGAAAGTAAAATATTCCAAAGACTGAGTGAAGTAAAGGAGAGACTATTGGCCAAATGTCTATCTT
NC_025343_Sosuga_2012_M_8829_8909	TCAAAGAAAACGAAAAAACCTGTCCTTAAATTTCAATCCTGTACCATGGCCGAAATGCATGACTATACTGCACAGAGTAA
NC_025343_Sosuga_2012_M_8910_8990	CATCAATTCGTCACTTGAAAGATTTAAAGATGCAGAACATATAATCAGTCATGCATTAGCACCGTTGTCACCTGGCCTCA
NC_025343_Sosuga_2012_M_8991_9071	AACCCTTCTAGAAGATGTGTCAAGCAAAATTACAGGGAAACAAAACCTATTCCAACAAAATATTAAGTCCTGTAGTTTTC
NC_025343_Sosuga_2012_M_9072_9152	CCCGAATCATTCTAGACCCAATTCTGTCATGCACATTGCCACAAGTGCATTCCAAGTGGAGTGGAAGGACAACTTCAATC
NC_025343_Sosuga_2012_M_9153_9233	ATGGTTTTTGATAAGGCATTTAATGAGACAGCTAATTATGAGGAATAGTGAGGGCCACACACAGATTACACCTGTAGAGA

NC_025343_Sosuga_2012_M_9234_9314	TGATGATAGGATTATGAGTGTGATCATTACACCAGAGTTAGTAACCATTTGGCTATCAGAGAACAACACAATCATTTATC
NC_025343_Sosuga_2012_M_9315_9395	GACTTTTGAGATGGTCCTGATGATCACTGATCTGTATGAAGGACGAATGAACATGATGCTCCTAACTACCTTAAGCTGTT
NC_025343_Sosuga_2012_M_9396_9476	TTTGGCACCGCTTAAATCAAGGCTAAAGCAGCTGTTCATCATTGTGGATGACTTAAGTTCCATCATAGGGAATTCAGTTT
NC_025343_Sosuga_2012_M_9477_9557	CTCCATTGTTGCATCTATGGAAAGTTTAGTGTATGGTCAACTCCAACTTTCTGATCCTGTACCTGATGTAGCAGGTGAAT
NC_025343_Sosuga_2012_M_9558_9638	CTATTCCTTTGTTGTCGGAGAAATGTTAACAGCACTGACAGATACAAAAGAATTCACAACAGATGAGGCTAGAATTGTAG
NC_025343_Sosuga_2012_M_9639_9719	GGAAAGAATTACTGAGTGTTATGAGGATTTAACACCGGACTTGATTGCTGAACTGCTCTGTGTCATGAGGATGTGGGGTC
NC_025343_Sosuga_2012_M_9720_9800	CCCATCTTTGAGTGCAGAAAAAGCTGCTGAAAAGGTGCGAAAATCTATGTGTGCTCCAAAGATAATAGATTTAGAAACAA
NC_025343_Sosuga_2012_M_9801_9881	TCTGAAGACACTTGCTTTCTTTCATGGAATATTGATCAATGGTTACAGGAGGAAGCATAATGGGATTTGGCCTAAATGTA
NC_025343_Sosuga_2012_M_9882_9962	ACTTCCACCAAATGCAGGAGTAAGCCTGACTGAGATGAAGCATGACAATTCTGAGCTCCCACATCAGTACATCCTAAATC
NC_025343_Sosuga_2012_M_9963_10043	TTGGAAAGAGATTGCTTTTATAGAATTTGAGAAAAGCTTCGATTCAGACCCAGGTGATGACTTGAGTATATTCATGAAGG
NC_025343_Sosuga_2012_M_10044_10124	TAAGGCTATTAGTGCACCAAAAAGTGACTGGATGAGTGTGTTCAGGAAAAGTCTCATAAAACCAACTTGTGAGCGGTTAC
NC_025343_Sosuga_2012_M_10125_10205	TGCGCCAATGCCAAGGCCATTTAATAGGCGGTTATTGCTAAACTTTTTGGCAGACAGCAACTTTGATCCATCCAAGGAGT
NC_025343_Sosuga_2012_M_10206_10286	AGAGTATGTGACAACTGGAGCGTACCTTGAAGATGAGGAATTTTGTGCCTCCTACTCACTCAAAGAGAAGGAAATAAAGG
NC_025343_Sosuga_2012_M_10287_10367	GACAGGAAGAATCTTTGCAAAACTAACAAAGAACATGAGATCCTGCCAAGTAATGGCAGAGTCACTCCTGGCCAATCATG
NC_025343_Sosuga_2012_M_10368_10448	AGGCAAGCTGTTCAAAGAGAACGGAGTTGTTCAAGATAATTTAAATCTTACCAAATCCTTGTTAACAATGTCCCAGATTG
NC_025343_Sosuga_2012_M_10449_10529	ATTGATCTCCAGACAGTCAAGACGAAACATTAGAGAGAATGTGACAATCCTTCACAAAGTCACAAAAAACCATCACCAAA
NC_025343_Sosuga_2012_M_10530_10610	GTTCTCATCTGCCCCGAGCACTAAAACTGGACAAAAGTCACAGCAAGACGAGCAGTTTGAGATTGCAGCTTTTTATCTGA
NC_025343_Sosuga_2012_M_10611_10691	TACAGATCTTGAGAAGTACTGTCTCAATTGGAGGTACCAGACTATAATTATGTTTGCAACATCAATGAATAGACTATATG
NC_025343_Sosuga_2012_M_10692_10772	TTATCCTCACTTATTTGAGTGGATTCATCCCCGACTAATGAGGTCAACACTGTATGTCGGAGACCCTTTTAATATGCCAC
NC_025343_Sosuga_2012_M_10773_10853	CTACCTTGATACAACTGATCTTGACTTAGTTAAGAATGAAGGTATTTTCATAGTCTCCCCCAGAGGTGGTATAGAGGGTC
NC_025343_Sosuga_2012_M_10854_10934	GTGTCAGAAACTCTGGACAATGATTTCCATATCTGTCATTATCCTTTCTGCAGCTGAATCCAATACAAGAGTAATGAGCA
NC_025343_Sosuga_2012_M_10935_11015	GGTACAAGGAGACAACCAGGCAGTCGGTATTACCACCAGAGTTCCAAGATGTGTAAGTACAAAAGAGAAGAAAAGGATTG
NC_025343_Sosuga_2012_M_11016_11096	TTTCGAGAACAGCAAGGCATTCATTGAAAGGTTGAGAGTGAACAATCATAATTTAGGGCACCACCTCAAGAGTCAAGAGA
NC_025343_Sosuga_2012_M_11097_11177	TATACTCAGTTCCTCTTTTTTTGTGTATAGTAAGAGAATTTTCTCAAATGGCAGGATACTTAATCAGGCTCTTAAGAACA
NC_025343_Sosuga_2012_M_11178_11258	GAGCAAAGCCAACTTAATATCTGATGTCTTAGGTGAGTGTACCCAATCTTCATGCTCAAACCTAACAACAACACTGATGA
NC_025343_Sosuga_2012_M_11259_11339	ATTGACTGAAAACGGTGTAGAAAAGGACATCTGTTACTGGCTCAGCTTTTTCCTTTCTGCTAAACAGCTTGTTTATGATC
NC_025343_Sosuga_2012_M_11340_11420	ACTGTTCCCTCTGCTAGCTCAATTCGAAGATGAAGTGACATCTACATACCTTAACAACCCACATCTGATCGGGAGACTTT
NC_025343_Sosuga_2012_M_11421_11501	TGCTATTCCCTCTCAACTGGGTGGTCTTAATTACTATTCTTTAAGTAGACTTTTCTGTAGGAATATAGGTGATCCCCTGA
NC_025343_Sosuga_2012_M_11502_11582	ATCTGCAACAGCTGACCTCAAGAGGCTGATTCAACATGGAGTTCTACCAGAGTGGTATCTCAATAATCTCTTCAAAAGGA
NC_025343_Sosuga_2012_M_11583_11663	ACCTGGAGATGGTAACTGGACTACCCTTGCAGCTGACCCTTATGCTTTAAATATTGACTACATTTACCCTCCAACCTCCT

NC_025343_Sosuga_2012_M_11664_11744	CCTGAAAAAACACACCCAAAGGGTTCTGATGGAACACAGCACGAATCCCATGTTGGCAGGTGTATTTGTAGAAAGTTCTT
NC_025343_Sosuga_2012_M_11745_11825	CAGCGAAGAATTAGATCTTGCAAAGTTCTTGCTAGACAGGCCCATGGTAATGCCGAGAGTAGCACACATTATTGTCGAAC
NC_025343_Sosuga_2012_M_11826_11906	GACATCATGTGGTCGGAGAAAGCAAGTACAGGGTTACCTAGACACCACAAGAACAATGATCAAACACGCCATTACAAAAC
NC_025343_Sosuga_2012_M_11907_11987	ACCACCGAGCTTCAGAAAATTAGAGAAAATTATAAATTATAATAAATTGTACTTGGGTTACAATTTGGAAATGATACAAA
NC_025343_Sosuga_2012_M_11988_12068	GCCAGTTGGGGGTAGACAAAAGATGATAGAGATAGCTCACATCTCAGATTTATGCAGTATAGATATCTCAAAGATATTAA
NC_025343_Sosuga_2012_M_12069_12149	GAGAATCTCTTGGTCGAGCTTGCTCACAGGGAGACCGTTGGAAGGTCTAGAAACACCCGATCCAATTGAACTCATTAATG
NC_025343_Sosuga_2012_M_12150_12230	GTCACTGATTGATGGTTGCAATATCTGTCACCATTGTTTATCTGGGGACAGGAAATTCACCTGGTTTTTTATCCCTTCAG
NC_025343_Sosuga_2012_M_12231_12311	GATAGAACTGGACACCCCCCCTGAAGACAACCCTCCAATGAGAGTCCCATATGTGGGTTCAAGGACAGATGAGCGGAGAG
NC_025343_Sosuga_2012_M_12312_12392	GGCATCAATTGGATATGTCAAAGGGGCTTCACCATCACTTAAATCGGCTCTAAGGCTTGCAGGCGTGTACATCTGGGCTT
NC_025343_Sosuga_2012_M_12393_12473	CGGAGATACTGAGAACAATTGGGAAGATGCACTGGATCTTGCAAATACTAGAGTTTATATTGAAATGGACCAACTAAAGA
NC_025343_Sosuga_2012_M_12474_12554	TCTCACACCAATGCCTACCAGTGCAAACCTCACACACCGATTGGATGATGGCTTGACTCAAATGAAGTTTACTCCTGCTA
NC_025343_Sosuga_2012_M_12555_12635	TTCGTACGCATATTCTTGTTTTGTGCACATCTCCAATGACAACCAAAATTTAGAACAACTAGACAGGTTGTTAGACTCCA
NC_025343_Sosuga_2012_M_12636_12716	TCTAATCTATCAGCAAGTGATGATTTTAGGTCTAGGGGTCATTGAGACCTGGCTATCATTTCCGAACCAAATTAATGATG
NC_025343_Sosuga_2012_M_12717_12797	AGACATCTCTGTCCACCTGCATACAGGGGGTTCTTGTTGCATCAGGCCAGTTGACATGTGTGTTCTAAATGAGAGTCCTA
NC_025343_Sosuga_2012_M_12798_12878	TGTACCTCCGACCTTGAATGTTCCACACCACAATAAGTTCATCTTTGATTCAGATCCTTTAGACAAAGATCAAATTGAGG
NC_025343_Sosuga_2012_M_12879_12959	AATTGATAATGTCATGATTCAGTCACGTTTATGTGGAATTGATTGTTTAACGATAGAAGAAAGAATCCCACTGCTTGCAC
NC_025343_Sosuga_2012_M_12960_13040	CCTAATTTCATTGCAATTTGCTCATACGCTGACTGGACTAGATGAGTCAACATCACTCATTAATGATGCTGTGGTAGATG
NC_025343_Sosuga_2012_M_13041_13121	TGATTATTCAGCAAACTGGATCAGCGAGTGCCTGAATACATATTTAGATAAAGTATTCTATTATACTGCCTGGAATATAA
NC_025343_Sosuga_2012_M_13122_13202	GCTGGACTTGGCATACCAGATGTACTACATGAGGATTATAGGGCTTCATTCCATAATTGACTACCTAAACATAGTTTTAA
NC_025343_Sosuga_2012_M_13203_13283	TAGGATCCCAGGGTTGGCACTGACTGGTATATCATCAACTATCAATCATCCCAAAATCTTAAGACGACTCATAAACTTGG
NC_025343_Sosuga_2012_M_13284_13364	AATTCTAGTCCCAAAGAACTCCCCCTATCTTGCAACACTGAACTATCATAAGCTTGCAACAGAAGCAATAATGTGGGGAG
NC_025343_Sosuga_2012_M_13365_13445	GCACCAGGCTCTAGCAGACATCAGGACTGGAGTTGATATTGAACTTATAATACCATCTGAAGATAGTACAGAATTGTCTG
NC_025343_Sosuga_2012_M_13446_13526	TAGAACACTGAACCTAGTTGCAAGGAAGTTAACTCTAATAGCAATTGCTATTGCAGATAATCCAAATTTGCCTTACGTGA
NC_025343_Sosuga_2012_M_13527_13607	GGGTTTAGCTCCTGATCAGAAGTGCAAGGTACTCACTGAATACCTACTAGTCCAGGTGAAGCTTTTAACTGCTGAACCAG
NC_025343_Sosuga_2012_M_13608_13688	GAAGCTTGCTATGTGGAAGACAGTAATCTGTGAGCCAAAAATGTCTGCATTCCCTCACAACCTGTACTATTTAACTAGAA
NC_025343_Sosuga_2012_M_13689_13769	GATTTTGAATTCGATAAGGGAATCACCACTGGGGCAGACCTTAATAGATCAATTTTATAGCAGTTTTGGTTTTTTAGAGT
NC_025343_Sosuga_2012_M_13770_13850	AGACCCAAACATGACCTTAATGAATCTGGCATCAAATTTGGGCGGTGAATTTCAGAGTCTAACTGTGTTCGATGTCTCCA
NC_025343_Sosuga_2012_M_13851_13931	GGATATTTATAAGGAGTCAGATGATAACGAGAGATACCACTATCCATTCCTGAGCCAGGACTCAGTGGATCAGACCGCAA
NC_025343_Sosuga_2012_M_13932_14012	ATTTGCAGCCCCGCCTACGCATCATATATTGAGGCCCATTGGACTCTCCTCCACCTCTTGGTACAAAGGAATTTCAATAA
NC_025343_Sosuga_2012_M_14013_14093	CAATTATTTGGCAAATAAAAGAGTTCCCCTGGGCAATCATCTTTATCTGGCAGAGGGTAGTGGGGCACTAATGACCATAA

NC_025343_Sosuga_2012_M_14094_14174	TGAAGCTTACTATCCAGGAGAGAAGATATATTACAACAGCTTCTTCTCAAGCGGCCAATGCCCCCCTCAGAGAAACTTCC
NC_025343_Sosuga_2012_M_14175_14255	ACCTCTTCCCACACAATTCACTGAGAGTATCATCTATCAACATCTAATTAATAATATTCCTTGTGAATTGGGCTTTGTTC
NC_025343_Sosuga_2012_M_14256_14336	AGAGTTCCTACCACTCTGGAGCGGAAATAGTAAACAGACTGACTTAAGCAAGAGAGAGTGTGTTGAGTTTATATTGAGTC
NC_025343_Sosuga_2012_M_14337_14417	AGTCTCCACAGGGACAGTGTCACTACTAGTATGTGATTTAGAGGAAGGGTTCATATCGAACAACTGTGATCTATCCTGTG
NC_025343_Sosuga_2012_M_14418_14498	ATTGATACATGTTTTACTATTATCTTTCATGTTAATAATGGATAATGGATTACTTATCCTAAAATCAGACCTAGAACCAT
NC_025343_Sosuga_2012_M_14499_14579	CCAAAAGCTGTCTACAATAGTATCCATCCTGTCATACAGGTTCAAAAATATTACCTTAGCAAGATCAGCATACTCTGACC
NC_025343_Sosuga_2012_M_14580_14660	TCAAAGTAGTGAAGTATTTCTGATTTGCCTTGCTGGACCAGAGAAAACCGTTGGGACATTCAATGTGGCAAGAGCCCTTG
NC_025343_Sosuga_2012_M_14661_14741	ACAACAGGATAAGATGGATGGGTTCTCATTGATAAATCCATGTGTTCTCAAGGATCTGCGGGAGGAGATGGCTGAATCAT
NC_025343_Sosuga_2012_M_14742_14822	CAATAATGTAATTGATGTGATCGATATGCAGCTTAAAATGGCACATGCACTTGAGAATCAAGACGATCATGTACTGCTAT
NC_025343_Sosuga_2012_M_14823_14903	AAGGCTAGGCAGCAGATCACAGACAAACCAGCTACTCCAAACACCACAAGCCAACACTATCCAAGAAGTCATTGAAAGAA
NC_025343_Sosuga_2012_M_14904_14984	AGCCAACTTGGTTACTATATACTTGAAGGAGACACTGACTACAATTGAATCTTACAATGAAGACAGAACCAGGCTCCTAT
NC_025343_Sosuga_2012_M_14985_15065	TCTAGCATATAATATTTCGGCTTTCGGAAAAATTAGAACATCAGCCCAATTGACAGCCAGGGCCGTACTGGATATCGTGA
NC_025343_Sosuga_2012_M_15066_15146	TAGAAACTGGCCTATAATTGATTTCCATATGAGGAAAGTTGTGATAGCTTCTTTGAAGTTGGGTGAATTCAGTGTAAGTC
NC_025343_Sosuga_2012_M_15147_15227	AGTTTTGACTCCAAAATCATTCCTCAGTATCACTCATGCCAGGAAGTATTTTTCACAAAACCTTGGACTTGACCAATTGG
NC_025343_Sosuga_2012_M_15228_15308	TATTGAACTAGGGCAAAACCTAAAAGTTCTTCTACCCAGATCAGAGCAAAAACTTATCTGGAAAAATATCGGCTGCATTA
NC_025343_Sosuga_2012_M_15309_15389	TTTGATATCTACTCTTGAAGATGATAATAATGATTTCAGGTATGAGCCATACCCAGATGAATCTCAGGACCAGCCCGAGT
NC_025343_Sosuga_2012_M_15390_15470	CGATCTCGCTGGCGATGAAATCTAATTGACTAGGAAGAGAAAATGCAGTCTCAGATATTTAAGAAAAAACTTATAGATTT
NC_025343_Sosuga_2012_M_15401_15480	GGCGATGAAATCTAATTGACTAGGAAGAGAAAATGCAGTCTCAGATATTTAAGAAAAAACTTATAGATTTTCCCCTTGGT
NC_025350_Tuhoko_virus_3_M_0_80	ACCAGAGGGAAAATTAAGAAGGGTCTTTCCAGTCAGATGAGATTCCTGATTCAGTGGGCCCGGAAGTGGCATAGTTCGGG
NC_025350_Tuhoko_virus_3_M_81_161	TCGATTACGACTCTGAATGGTCTCTTCCGGAGCTTTAATTGGATAATTCGGTATCGTTGAGGTACAACCAAATTATAGCA
NC_025350_Tuhoko_virus_3_M_162_242	TCATGGCATCACTCTTCAGAGCCTTGGAACAATTTACTCTGGAACAAGATCAAGGCGGTCGCGGTAATGTGGGTGACCAG
NC_025350_Tuhoko_virus_3_M_243_323	CGCCAGAGACTCTCCAGGCAACTATCAAAGTCTTTGTAATTAATACACCTGATCCCCGCTTAAGATATAGGATGATGTGC
NC_025350_Tuhoko_virus_3_M_324_404	TTTGCTTGCGCCTAATAGTTAGTAACTCTGCAAGGACTGGGCAGAGACATGGAGCTCTCATGACACTGCTCAGCTTGCCA
NC_025350_Tuhoko_virus_3_M_405_485	CTGCAGTGATGCAAAATCATTTTCGCACTGCAGAGCGATCTCCTGACTGCCAAATTGAACGGATTGAAGTTGATGGATTT
NC_025350_Tuhoko_virus_3_M_486_566	AACCGGGGACTTACAGAATCAGAACAAATGCAAGAACCCCCCTGACCCATGGAGAGGTGGTCGCCCTTGAGGAGATGGCA
NC_025350_Tuhoko_virus_3_M_567_647	ATGACATCCCAGAAGCTCTAGCAAACCACACACCCTTTGTGGATGCGCAGACTGAACTCGAAGAATGTGATGAGATGGAA
NC_025350_Tuhoko_virus_3_M_648_728	AATTCCTTGAGGCGATCTACAGCACCCTCATCCAAGTGTGGATAATGGTTACAAAGAGCATGACCGCATTTGACCAACCA
NC_025350_Tuhoko_virus_3_M_729_809	CAGGGTCAGATGAGCGTAGGGTTGCAAAATACCAGCAACAAGGCAGGCTTAACCCTCATTACCTTCTGCAAGGAGAGGTG
NC_025350_Tuhoko_virus_3_M_810_890	GTAGGCGGATCCAACTATGCATAAGGCAGAGCCTCCCCATTAGGCAATTCCTTGTGAATGAATTGCAAACAGCAAGCAAC
NC_025350_Tuhoko_virus_3_M_891_971	AAGGTCCAATTACAGGGAAATATTATGCAATGGTGGCGGACATCGGGAAGTATATTATGAATGCTGGAATGGGGGGATTC

NC_025350_Tuhoko_virus_3_M_972_1052	TCATGACCATCAGATTTGCTCTTGGACAAAAATGGCCGCCACTTGCACTAGCTGCATTCTCGGGAGAGATAGTTAAGATG
NC_025350_Tuhoko_virus_3_M_1053_1133	AGAGCCTAATGCTTCAATATAGAAGATTAGGAGACAGGGCAAAATTTATGGCACTGTTGGAAATGTCAGAGATGATGGAT
NC_025350_Tuhoko_virus_3_M_1134_1214	TTGCACCATCCAACTTCCCACTATTGTATAGTTATGCAATGGGTATAGGGAGTGTCCAAGATCCCCAAATGAGGGGCTAC
NC_025350_Tuhoko_virus_3_M_1215_1295	CATTCGGAAGACCCTACCTCAATGCAGCATTTTACCAGCTGGGTGTCGAAACTGCAAACCAACAGCAAGGATCTGTAGAC
NC_025350_Tuhoko_virus_3_M_1296_1376	AAGACATGGCTGCTGAACTTGGGCTAACCGAGGCAGATAAGAGGGCAATGGCAGCAACTGTCACTCGACTCACGACAGGC
NC_025350_Tuhoko_virus_3_M_1377_1457	GAGGAGGGCTGCAGGGCAACATGGGTATCAATGCCATGGCAAGAAGAGGGCAGCAACTCCCGCAGCCTCAAGACAATGTA
NC_025350_Tuhoko_virus_3_M_1458_1538	TAGAAGAAGATGAGGAAGAGGATGAGGAAGAGGAAGAACCCCAGAATCAAGAGAGGGAGGTAGATCCAGAACTAGAAAGG
NC_025350_Tuhoko_virus_3_M_1539_1619	GAGTGGCACTCAGGATCGCACAGGATCAAGAGAGGTGGGCCAGGAGGTTGGCTGAAATAGAAGCAGAACGAGTTGCCAGA
NC_025350_Tuhoko_virus_3_M_1620_1700	AAGCAGCCAGCCATCCACCTCAACATGATGCAACAAGAGATCAAATACCTGATCAACAAGAATATGATAGCAACCTAATT
NC_025350_Tuhoko_virus_3_M_1701_1781	AGTAGCAAGGCTTACAAACAACGAGCGGGCTTGCGCAAACTGCATCGCAATATATTATGTAGGAGCTTTATGTCTTTAAG
NC_025350_Tuhoko_virus_3_M_1782_1862	AAAAACTATAGTAGGCCCGAAGGTTGGCATCTTCCGGGCTCTCGCTGACTTCGGCTCCGAAGTCTCTTCTGCCACTCAAC
NC_025350_Tuhoko_virus_3_M_1863_1943	TAGGACTGATTTTGAAATTGTTACCACAACTTCCCAATATGGAACCCACTCCGTCAGACGCGGAAATCTCCGCATGGATA
NC_025350_Tuhoko_virus_3_M_1944_2024	AGAAAGGACTAGCTACTGCCAAGCATTTTGCTCCAAATCCCGTATCATCACAAAGCAGCTTAGGGAAATCAACAATCAAG
NC_025350_Tuhoko_virus_3_M_2025_2105	AGGGGAATACCAAAGTCCTCGTCTCATCAGCAGAACAAATTGCCAGCTCCCAACCCGCAGCCAGTCACACCGTCAAGGTT
NC_025350_Tuhoko_virus_3_M_2106_2186	AAGCTCAAGTCCACCCGCAACAGCCCACCGCTTCCCAACCATGCACTGGAGCAAGACCAAAAACAAAGAGAACTGCAACA
NC_025350_Tuhoko_virus_3_M_2187_2267	CTGCCCCGACCCCGTCTGTCCAGCAGGCAGTCAAAATAGAGCCTGTATATGAGGATATTGTATCAAATCCCACTCATCAA
NC_025350_Tuhoko_virus_3_M_2268_2348	CTGAGAATGCACCATTGATTGCAACTCAATCATCTGCAAAGCAGTCACTGCTGTGTACTGAGCCTCTTCCCAGCCAACCT
NC_025350_Tuhoko_virus_3_M_2349_2429	CTTCCGCTACTCGCTGTGGAGAGCAAAGCTTTAAGAGGGGGCGATTTTCTTCCTGTTCCACCGGGAAGAGATCCAACAGT
NC_025350_Tuhoko_virus_3_M_2430_2510	ACCCAGGACACAGACGAGAATTTGATCTTGTTTGGGGCTCAGGAAAATTTGAGGTCAGAGAATGGTGCAACCCAACCTGC
NC_025350_Tuhoko_virus_3_M_2511_2591	CCCCAATCACTCCAATCCCCAGAAGATATCAGTGCCGTTGTGGGGAATGCCCTAGAATCTGCAAACAGTGTGAGGGAGAT
NC_025350_Tuhoko_virus_3_M_2592_2672	ATCAGGTACTTGAAAGTCATGGAGGCCAAAATGACACAAATTGAATGGAAAGTGGATAAAGTACTGGCTCAGAATAGTCT
NC_025350_Tuhoko_virus_3_M_2673_2753	ATCCAGCAAGTCAGGAATGAACAGCTGGTGCTCAAAGCTAGCATGGCAACAATTGAAGGATTAATGACAACAATAAAAAT
NC_025350_Tuhoko_virus_3_M_2754_2834	ATGGATCCTGGGGTAGGGCCGGGCGCAACAGCCGCACAAGCCAAAAGATTGTTCAAGGAAGCTCCGGTAGTAGTCAGTGG
NC_025350_Tuhoko_virus_3_M_2835_2915	CCCATAGTAGGTGACAATGATCTCATCTTTGAGGACAAAATTGAAATCAGTAGTCTTGGGAAACCACAAAAGGTTGCTCC
NC_025350_Tuhoko_virus_3_M_2916_2996	CAACCAAAGAAGCGGCTTGTCACTAGTGAGGCTGACATAGCTGGTTATAAACTCACTCTCACAAAATTACTAAAAGAGTG
NC_025350_Tuhoko_virus_3_M_2997_3077	ATTCCCAACGCCAATCAGCACAAAAAATTTGAAGATCTGATAGCTAGTGTAAAGAATGAAAGTGATTTCAAAGCAGCAAA
NC_025350_Tuhoko_virus_3_M_3078_3158	AGGGAAATCGTGAGAGCTGCAATCTAGAGATCAGTCAACACCAACTTACAATCTTACCTATTAACTTTAGCTTGTGCCTT
NC_025350_Tuhoko_virus_3_M_3159_3239	AACCTATAATCTGCTTCTGCATAGCATCTAATCTCTCAAAACAAAAAGCATTCCTACTTCGAAACTCACCTGCCAACACA
NC_025350_Tuhoko_virus_3_M_3240_3320	AAAAAAATCATAGCCTGAAGGCCTTTAAGAAAAAACTAGGCTGGGGTCGAACCCATAGACCCCTAACGTCCCCAACAATG
NC_025350_Tuhoko_virus_3_M_3321_3401	CTGGGAGACAGGCTACAATTCCTGTTCCAATCAATTTTGAGAGCCCAAAGAACTATCTCAATGCATTCCCAATCGTCCAA

NC_025350_Tuhoko_virus_3_M_3402_3482	CTGACCCGTCTGTATCTGGAGAAGCCGGGAAGCTGCTCAAACAAATCAGATTCAAAGATCTAACCCTTAGGGGATCCACC
NC_025350_Tuhoko_virus_3_M_3483_3563	AAGCCCCTATCTCATTTGTTAACTCATATGGATTCATAAAGCCATTGCGCACTAGGGAGGAGTTCTTTTCTGAAATGCAC
NC_025350_Tuhoko_virus_3_M_3564_3644	AACCATCCCAAGCCCCCTGTCTTACAGCTTGCTGCCTCCCTTTTGGAGCTGGTCCGGCCATAGAACACCCAGATAAGATA
NC_025350_Tuhoko_virus_3_M_3645_3725	TGGATGACCTAGACAAAGTGTTTATAGTGGTTAGAAAGAGTGCAAGTACTATTGAGGAGTGTGTGTTCGATATCCGGAAA
NC_025350_Tuhoko_virus_3_M_3726_3806	TGCCATCCAGCCTATCCCGCCACCAGCTTGCAGGCAACCGTGTTCTTTGTGTTGCATCGGATAAATACATCAAGGCTCCG
NC_025350_Tuhoko_virus_3_M_3807_3887	GTAAGCTGACTTCAGGTATGGATTATACATACAACATTGTCTTTCTCTCTATAACTCACTGCCCGCCAAGTCAAAAATTC
NC_025350_Tuhoko_virus_3_M_3888_3968	GTGTTCCTATGCCTATACAGAGCCTTAGAGCTAAGGTGATGAGGAGTGTCCACCTAGAAATTATGATTAAAGTTGACTGT
NC_025350_Tuhoko_virus_3_M_3969_4049	ACAAAAACTCACCGATCACCAAGAATCTGATCTATGACCCAAGTAATGACATCTGGATGGCATCTATTTGGTTTCATCTG
NC_025350_Tuhoko_virus_3_M_4050_4130	GCAATTTCTACAAGGGTTCAAAGCCGTTCAAGGAATATGATGATCAGCACTTTGCCTCAAAATGCAGAGCCATGGGTCTA
NC_025350_Tuhoko_virus_3_M_4131_4211	AAGTAGGCTTAGTAGATTTTTGGGGTCCAACATTTTTGGTCAAAGCACATGGTAAGATTCCTCACGCAGCAAGACCTTTC
NC_025350_Tuhoko_virus_3_M_4212_4292	TTGGTAAGCACGGATGGGTCTGCCATCCAATGATGGACTGTGCTCCTGCAATCAGTAAGTCACTTTGGGCATTGAGTATA
NC_025350_Tuhoko_virus_3_M_4293_4373	CTATCCTACAAGTCAACGCTGTTTTGCAAGCCTCAGACTTAAATCAGATGATTAGAATGACCGACGTTGTGTTCCCAAAG
NC_025350_Tuhoko_virus_3_M_4374_4454	TGAAGATTAACCCAGATATTGCTGGAGTACAGAAGACTCGCTGGAACCCGGTAAAGAAATTAGTCACCATTGATTAGCCA
NC_025350_Tuhoko_virus_3_M_4455_4535	AAATCTGCGCATCCTGTATGGCACTGGAACGCCGTCCCCACATCAGTCAACCCCAGAAATCAAAGACATACACATTTCCC
NC_025350_Tuhoko_virus_3_M_4536_4616	CCCAAAACAAACTGTAGTTAATAATCAACCCCATCCCATCTGAATGATCATTCTCCTTAATCACGCATATACCTTCAGTT
NC_025350_Tuhoko_virus_3_M_4617_4697	AAGAAAAAACTGCAAAGAGTCTAAGACTTGGAGGAAATGAAAGACGGGGGCGAACCCATACACGATAGACCCCACAGCCA
NC_025350_Tuhoko_virus_3_M_4698_4778	AGAACTGTCCATTATAGTTAAATGAACAATGCTCTGGTTAACTATCTTAATTGCCTTAGTTGGGAATCATGAGTCTACTT
NC_025350_Tuhoko_virus_3_M_4779_4859	CATGAATATAAATTTTCTACAATCTTTAGGGCAAATAAATTCCCAAAAGAGATTTCTGAATTTCTATACCCAACAACCAC
NC_025350_Tuhoko_virus_3_M_4860_4940	AAGTTATATGGTAATCAGGTTGGTTCCAACTCTACAACTTTCTGCTAACAACTGTACTCTTGGTAGCATAGTACGTTATC
NC_025350_Tuhoko_virus_3_M_4941_5021	TAATGCTATTAAGGAGCTAATACAACCCATGGATGAAAATCTCAGATGGTTATCATCAAATCTTATTCCACAAAGGCGCG
NC_025350_Tuhoko_virus_3_M_5022_5102	GAAAAGATTTGCTGGAGTGGCTGTAGGTTTAGCTGCTTTAGGTGTTGCAGTTGCTGCTCAAGCAACTGCGGCAGTAGCAT
NC_025350_Tuhoko_virus_3_M_5103_5183	GGTGGAAGCTAGAGCAAATGCAGAAAAAATAGCATCCATGTCTCAGTCAATACAAGAAACTAACAAAGCAGTAACATCTC
NC_025350_Tuhoko_virus_3_M_5184_5264	TAGTCAGGCAGTATCTGCAAGTGGCATTGCTATACAAGCCATTCAAAACGAGATCAATAATGTGATTCATCCAATACTAA
NC_025350_Tuhoko_virus_3_M_5265_5345	CCAAGTCCAATGTGATGTATTAGATGCCCGGGTAGGCAACATACTTAATCTATATTTAATAAAAGTCACAACAATATTTC
NC_025350_Tuhoko_virus_3_M_5346_5426	AAATCAGTTAACAAACCCTGCTCTACAGCGATTATCAACTCAAGCTCTGAGCATGCTTATGCAGTCTACTTCATCTTATC
NC_025350_Tuhoko_virus_3_M_5427_5507	GCGTAATCTTTCATCCTCAGAAAGTGCAATTAATGCAGATTTAAGTATGACCAACTTAATCGAAGCCCAAATAGTAGGTA
NC_025350_Tuhoko_virus_3_M_5508_5588	CAATATGACAAATCTTCAATTAGTCCTTGCTGTCTTCATACCTTCTATTGCGAGATTGAATGGCGCGTTGCTCTATGACT
NC_025350_Tuhoko_virus_3_M_5589_5669	TATTAGTATTACAATCAGCTCAAATCAAACCGAGGTTATGCTACAAATTCCTCATAGGGTTTTAGAGATAGGGAATAGTC
NC_025350_Tuhoko_virus_3_M_5670_5750	TTATACATTTGAAGGTACACAGTGTGAGATGACTAAGCTAAATGCTTATTGCTTGTACAGCGATGCCATTCCAGTAACTG
NC_025350_Tuhoko_virus_3_M_5751_5831	ATCTCTTAGAGACTGTATGAACGGGTTGTTTAGTCAATGTGGATTTGTAAGGATAATTGGTTCTTTCGCTAATAGATTTG

NC_025350_Tuhoko_virus_3_M_5832_5912	GAGTGTGAACGGGGTAATATATGCAAATTGCAAACACCTTACTTGTTCTTGCCTACAGCCTGATGAAATTATCACTCAAG
NC_025350_Tuhoko_virus_3_M_5913_5993	CACTAATGTCCCATTAACTATAATAGATACAAAGAGATGTACAAAAATAAGTTTAGGGCATCTGACATTTACCATAAGAG
NC_025350_Tuhoko_virus_3_M_5994_6074	GTACGCAAATGTAACTTACTCACTCAGGACTGAAATAGCAAATTCTCAAATCACTGTAGTGAGCCCTTTGGATTTATCTT
NC_025350_Tuhoko_virus_3_M_6075_6155	ACAATTGACCACAATAAACAATTCACTTGCTGATGCAACAAATCATATTATGAATAGTGATCGTATTTTGGATAGGCTCA
NC_025350_Tuhoko_virus_3_M_6156_6236	CTCTGGTCTGTATAGCAAATGGGTTATAATATTTCTTATATGTGCATCAATTGTATCACTAATAGGACTAGTATTTCTGG
NC_025350_Tuhoko_virus_3_M_6237_6317	CTTCCTAATTAGAGGGCTTATTTTGGAATTAAGATCAAAACACAGATCTAATCTTAATAAGGCTAGCACTTACTCTATTG
NC_025350_Tuhoko_virus_3_M_6318_6398	TTCTTCAATAGGGTTGACATAAATTCTCATCAGTGTCATTTTAATGTCTCATTAACTGTGATTAGTATATAGCTCTATGT
NC_025350_Tuhoko_virus_3_M_6399_6479	ATAGGTTAGATATGATTTTTAATAAAAAACTTGACATTGGAAGCCCTACCCGGGCCCGACCCAGTCAGCCCTCCAGCCCC
NC_025350_Tuhoko_virus_3_M_6480_6560	CAGCTCCACACACGGACCCACGACGACATCCAATCATGCACAACAGGACACAATCTGTCAGCAGTATAGACACCTCATCT
NC_025350_Tuhoko_virus_3_M_6561_6641	ATGTCTATCTCCCTCGGAGAAAGAAGGCCGTCACAAAATTCACATTTAAGAAAATCTTCCGAGTTCTAATCTTAACACTC
NC_025350_Tuhoko_virus_3_M_6642_6722	TACTATCAATTATTATAATCATAGCAGTAATCTTCCCAAAAATTGACCACATCAGAGAAACATGCGATAACAGTCAAATA
NC_025350_Tuhoko_virus_3_M_6723_6803	TGGAAACAATTACCAATCAAAACTCGGAAATCAAGAACCTGATTAACTCTGCTATCACTAACCTTAATGTATTATTAACA
NC_025350_Tuhoko_virus_3_M_6804_6884	CGACTACAGTTGATTTACCAATTAAGCTTAATAATTTTGGAAAGAGTATTGTGGACCAGGTAACAATGATGGTCAGACAA
NC_025350_Tuhoko_virus_3_M_6885_6965	GTAATGCTGTCTGTCGGGGCCCTGGAGATCGTCCGACTCAAAATATTGAATTATTCAAAGGTTTATACCACACCTCCCCA
NC_025350_Tuhoko_virus_3_M_6966_7046	CCTCAAATACTTCCACCAAATTGAGCATGATTACTGAAGCATCAAATCCAGATGATATAGTTCCCCGGCCCGGGAAACTG
NC_025350_Tuhoko_virus_3_M_7047_7127	TTGGCTGCACCAGGTTTCCATCTTTTAGTGTCCATTATGGGTTATGGTGTTATGGTCACATGGCTTCTACAGGGAATTGC
NC_025350_Tuhoko_virus_3_M_7128_7208	GCGGGTCATCTCCATCAGTGCAGATCATCAGAATAGGTTCAATTGGGACCAATAAGGACGGCACGCCCAAATATGTAATC
NC_025350_Tuhoko_virus_3_M_7209_7289	TAGCCAGTGCATCACTCCCAGAGACAACTAGACTGTATCATTGCTCTGTCACAATGACATCTATTGGATGCTATATTTTA
NC_025350_Tuhoko_virus_3_M_7290_7370	GCACTACTCCCTCAGTCTCCGAGACAGATGACTATAGTACTATGGGTATAGAGAAAATGAGCATATCCTTCTTATCATTG
NC_025350_Tuhoko_virus_3_M_7371_7451	ATGGATACTTAACACAGTTGGGTCAGCCAACTGGATTGGACAATCAAAATTTGTATGCTTTATATCCCGGTCCTGGGTCA
NC_025350_Tuhoko_virus_3_M_7452_7532	GTGTGATCTTTAGGGATTTTTTGATATTCCCGATGATGGGCGGAATTCGGTTAATGGATGCACAGAAGATGCTCAATAGA
NC_025350_Tuhoko_virus_3_M_7533_7613	ATATCACCTATAGAGGCTTTCCCCCAAGTGAGACCTGCACTGAAAGTGAACTTAAGCTTAAACAGGAAGTAGCAAATATG
NC_025350_Tuhoko_virus_3_M_7614_7694	TAACAAGTCCTTATTATGGTGAAGTGTTAGTGCTCAACTTCCTCTATGTATGCTCTCTCTTAGATAATATCCCTGGAGAT
NC_025350_Tuhoko_virus_3_M_7695_7775	GTTCTGTCCAATTGATTCCGCCGGATAACATGACACTTGGAGCAGAATCAAGGCTGTACGTATTAAATGGATCACTAATT
NC_025350_Tuhoko_virus_3_M_7776_7856	TGTACAAAAGAGGGTCATCATGGTGGCCGTATACAGAGCTATATCAGATAAATTATAGGGTAAACAATCGTGCATTCAGG
NC_025350_Tuhoko_virus_3_M_7857_7937	TGAGGGAATCCGTGAGGATTAATACAACATCCACAAGCAGACCAGGAGTACAAGGGTGCAATCTTGAAAAAGTTTGCCCA
NC_025350_Tuhoko_virus_3_M_7938_8018	AAGTTTGTGTATCAGGCATCTATCAGAGCCCCGGCATCATATCAGCACCGGTGAATCCAACAAGACAGGAAGAAGGGCTC
NC_025350_Tuhoko_virus_3_M_8019_8099	TTTATTTCCTTGTCTGGACTTCCTCCATGAGTTCGAGGACAGGACCACTGAGCTCATTATGTGATCATTCTACCTGTAGA
NC_025350_Tuhoko_virus_3_M_8100_8180	TCACATATCCCATTGGAGATGATACAATTTTCATAGGATATACGGATTCCAGTTGTTTCATGTCTAGTATCAAGGAAGGA
NC_025350_Tuhoko_virus_3_M_8181_8261	TTTATTGTATTGCCTTCTTGGAACTAGATAACCAACCTTACTCCATGATGGCTATTAGGTCTTTATCATATATTATCAAT

NC_025350_Tuhoko_virus_3_M_8262_8342	AGTAGAATTGGCCATCAGACTGTTACCCCTTATCTTACAAATTCTCTGCTTGGATTGAGCTTGATTACTTTAAGAAAAAC
NC_025350_Tuhoko_virus_3_M_8343_8423	ATGGGCCAGAATGGCTGCCACATCGCAGATCATTCTCCCCGAGGTCCATTTGGATTCTCCAATAGTAGAAAATAAGCTTT
NC_025350_Tuhoko_virus_3_M_8424_8504	GTATCTCCTCCAGCTAGGTGAGTTGCCAATCTTTGATTCTTACTATGAACATGACTTCTTCCCTGAAGTTGACTGGCCGA
NC_025350_Tuhoko_virus_3_M_8505_8585	AGTCCGAAGAGAGGAAAACAAGCTATTTTCTAGACTCAACGAGGTCAGAGATAGACTCTTGAGCAAAACTCTTTCCGCTA
NC_025350_Tuhoko_virus_3_M_8586_8666	TACTCGCAAGCTACACCACTCGCTACTGCCAACTCCAATCCCATGGCCCAGATGCATGCCCGAATTACACAGTGTTACCA
NC_025350_Tuhoko_virus_3_M_8667_8747	CAAGCACTCTTTGCAACGATTCACGGATGCTGAGCTTACCATCACACATGCCGCCGAAAGTATCAGTCAAGGTTTGTCTA
NC_025350_Tuhoko_virus_3_M_8748_8828	TGTGATTAATGAACTAGCAGATAAATTAACAGGTAAATCAGATCTATTCTCAAGGAATATTCAATCATGTATGGACCATT
NC_025350_Tuhoko_virus_3_M_8829_8909	AAGCCACACCCGCCCAAATGGACTGATTGACATTGCCCAAATTGCTCAAAGTACCGACTGGAAGGACAACTTTAATCAGT
NC_025350_Tuhoko_virus_3_M_8910_8990	GTTCCTGATTCGCCACTTAATGAGACAATTGATCATGGACAACAATACAGGGAAGATTTCCATTCCACCAATTGAGATTG
NC_025350_Tuhoko_virus_3_M_8991_9071	TGATAGACAGATGTCTGTAATTATCACTCCAGAGCTAGTCACAATCTGGATAGGGAAAACTAATCATGTATTCTACTTCA
NC_025350_Tuhoko_virus_3_M_9072_9152	ATTTGAGATGACATTGATGGTCTGTGACATGTATGAGGGCAGGATGAATGCAATTATGCTTACCACCATGAGTTGTTACT
NC_025350_Tuhoko_virus_3_M_9153_9233	GTCACCTTTAAGAAACAGGCTTCAAAGATTATACTATCTAGTTGATAGCCTGTGTGAAATCATAGGAAATAATGTGTATT
NC_025350_Tuhoko_virus_3_M_9234_9314	TATTGTGGCATCAATGGAAAGCTTGGTATATGGGAAGATACAACTTGCAGACCCAGTACCTGATGTTGCTGGTGAGTTCT
NC_025350_Tuhoko_virus_3_M_9315_9395	CTCCTTCATTATCACTGAGATGTTATCAGCATTAATTGAAACCAAGATGTTTACAATACAAGAGGCAACAACTGTCGTAG
NC_025350_Tuhoko_virus_3_M_9396_9476	GAGAATATCTGAATGCTATGACAATCTCTCACCTGACTTAATTGCAGAGCTTTTATGCCTTATGCGTATGTGGGGACATC
NC_025350_Tuhoko_virus_3_M_9477_9557	TTCCTTGAGTGCAGAGAAAGCTGCAGATAAAGTGAGGAAATCAATGTGTGCGGCAAAAGTAATTGATCTTGAGACCAACT
NC_025350_Tuhoko_virus_3_M_9558_9638	AAAGACACTTGCATTCTTCCATGGTATCCTTATTAATGGGTATCGCCGGAAGCATAATGGAATTTGGCCAAAATGTACCC
NC_025350_Tuhoko_virus_3_M_9639_9719	TCCACCCAATGCAAGCCTGAGTCTTGCCGAATTGAAACATGACAATTCAGAATTACCTCACCATTATATATTGCAACATT
NC_025350_Tuhoko_virus_3_M_9720_9800	GAAAGAGGTAGCATTCATCCAGTTCGAAAAGAGTTTTGATGCAGATCCTGGAGAGGAGCTCAGCATATTCATGAAGGACA
NC_025350_Tuhoko_virus_3_M_9801_9881	AGCCATCAGTGCCCCCAAAAAAGACTGGATGAGTGTCTTTAGGAGAAGCTTGATAAAGCCAATCTGCGAAAAGCTAGGTG
NC_025350_Tuhoko_virus_3_M_9882_9962	CCCGCTTCCAAATGCTTTTAATAGAAGACTACTGCTGAACTTCCTTTCTGACAGTAACTTTGACCCAGACAAGGAACTTG
NC_025350_Tuhoko_virus_3_M_9963_10043	ATACGTAACAACTGGGAGATACTTAGATGATGATTCATTTTGTGCATCTTACTCATTAAAGGAGAAGGAAATAAAAGAGA
NC_025350_Tuhoko_virus_3_M_10044_10124	AGGAAGGATTTTTGCAAAACTTACTAAAAATATGAGATCTTGTCAGGTGATGAGTGAGTCACTATTAGCTAATCATGCTG
NC_025350_Tuhoko_virus_3_M_10125_10205	GAAATTATTCAAAGAGAATGGTGTGGTCTTAGACAATCTTAATTTGACTAAATCTCTCCTGACCATGTCTCAGATTGGCC
NC_025350_Tuhoko_virus_3_M_10206_10286	GATATCAAAACAGTCACGGAGAAATGTTCGGGAAAATGTAACAGTGATGACCAAGGTGCATAAGAGAAGCAACTTAGCAA
NC_025350_Tuhoko_virus_3_M_10287_10367	CACAAAAATTCAGTCTAGTCATGGACCATCATCCCCTACAACTGATGAACAATTAGAAATTGCTGCCTTCTTTTTGACAA
NC_025350_Tuhoko_virus_3_M_10368_10448	TGATTTAGAAAAGTACTGTCTAAATTGGAGGTATCAAAGTATTGCAATGTTTGCCAATTCTATGAACCAACTGTATGGAT
NC_025350_Tuhoko_virus_3_M_10449_10529	TCCCCATCTTTTTGAGTGGATACACCCACGCCTGATGAGATCTACACTGTATGTGGGTGATCCATATAACCCACCCAGAA
NC_025350_Tuhoko_virus_3_M_10530_10610	CATCCACAGCACAGATCTAGATGAGATGAAGAACGAGGGAATTTTCATCGTATCCCCGAGAGGCGGGATAGAAGGGCTAT
NC_025350_Tuhoko_virus_3_M_10611_10691	TCAAAAACTGTGGACAATGATATCAATATCAATCATAATCCTCTCAGCAGCTGAATCTGGTACACGAGTGATGAGTATGG

NC_025350_Tuhoko_virus_3_M_10692_10772	GCAAGGTGACAACCAGACAATTGCCATAACAACAAAAGTTCCAAGAAGTATCCCTCATAAGGACAAAAAAAGGATAGCTT
NC_025350_Tuhoko_virus_3_M_10773_10853	TGAAAATAGCAAAGCATTCATTGAGAGACTAAGAGTAAACAATCACCATATGGGCCACCATCTAAAGAGTCAAGAAACTA
NC_025350_Tuhoko_virus_3_M_10854_10934	ATTGAGTTCCAATTTCTTTGTTTATAGCAAGAGGATATTTATCAATGGAAGGATTCTAAACCAATCCCTAAAGAACATGA
NC_025350_Tuhoko_virus_3_M_10935_11015	CAAAGCAAACCTCATATCAGATGTATTAGGTGAGTGCACTCAATCATCTTGCTCCAATCTAACTACAACAATTATGAGGT
NC_025350_Tuhoko_virus_3_M_11016_11096	GACCGAAAATGGGGTTGAGAAAGATATCTGTTACTGGCTAAGTTTCTACCTTTCAATCAAACAATTGACCTTTGATCTAC
NC_025350_Tuhoko_virus_3_M_11097_11177	GTTCCCCTTGACATCTCAATTTGAGGACCCGGTCACAGCAGCTTACCTCAATCATCCACACTTAATTGGGAGAATCTGTG
NC_025350_Tuhoko_virus_3_M_11178_11258	AATCCCTTCTCAATTGGGAGGTCTTAATTACTATGCTATGAGCCGTCTCTTTTGTAGAAACATTGGAGACCCTCTAACAT
NC_025350_Tuhoko_virus_3_M_11259_11339	TGCAACATCTGACCTCAAGCGCCTCATAACACACAAAGTGATCCCAGAGTGGTATCTAAGCAATCTTATAAATCGACATC
NC_025350_Tuhoko_virus_3_M_11340_11420	CGGAGAAGGCGGCTGGAACACTCTTGCATCTGACCCTTATGCACTGAATATTGATTACATCTACCCTCCTACTTCATTCC
NC_025350_Tuhoko_virus_3_M_11421_11501	AAAAAGACATACTCAAAAAGTGCTTATGGAGAACAGCATCAACCCCATGTTATCGGGTGTCTTTTCAGAGAACTCCAGTG
NC_025350_Tuhoko_virus_3_M_11502_11582	TGAAGAAGCTGCGTTAGCAAGGTTCTTGCTTGATCGGCCGCTTGTTATGCCAAGAGTTGCACACATTGTGATTGAACAGA
NC_025350_Tuhoko_virus_3_M_11583_11663	ATCTTGCGGCAGAAGGAGACAGATCCAAGGCTATCTTGACACAACTAGAACTATGATGAAGCATGCTCTAAATAAGCAAC
NC_025350_Tuhoko_virus_3_M_11664_11744	TCCTGGCTACCAAAAAGTGGAGAGAATAATTGAATACAATCGCCTATACCTAAATTACAATTTGGAAATTATTAAGAAAC
NC_025350_Tuhoko_virus_3_M_11745_11825	AATCAAGAAGAAAACAAAAATAATAATCAAGGCATCTATGCTTGACCTGTGCAGTATTGACATTTCAAAGCTATTGCGGA
NC_025350_Tuhoko_virus_3_M_11826_11906	ATTGTCATGGTCTCCACTTCTAGGTGGTCGACCGCTTGAAGGATTGGAAACACCAGACCCGATCGAGTTAATCTTTGGAT
NC_025350_Tuhoko_virus_3_M_11907_11987	GTTGGTAGATGGTTGCAATATCTGTCACTATTGTTTGGCAGGTGATAAAAAGTTTACCTGGTTCTTTATACCTTCGGGCA
NC_025350_Tuhoko_virus_3_M_11988_12068	CCTTCTTGACTCTCCACCAGAAGACAACCCGCCAATGAGAGTGCCTTATGTCGGGTCGCGCACTGATGAGCGTAGAGTTG
NC_025350_Tuhoko_virus_3_M_12069_12149	TTCAATTGGTTATGTGAAAGGAGCATCTGTCTCTCTAAAGTCTGCCCTACGCTGTGCGGGCATATACATCTGGGCATTCG
NC_025350_Tuhoko_virus_3_M_12150_12230	TGACACAGAAACGAATTGGGAGGATGCACTAGAGTTAGCCAACACCAGAGTGAATATTAACATGGATCAATTAAGAACTC
NC_025350_Tuhoko_virus_3_M_12231_12311	CACTCCAATGCCAACCAGTGCAAACTTAACTCATCGGCTTGATGATGGGCTATCTCAAATGAAATTCACACCTGCCAGTT
NC_025350_Tuhoko_virus_3_M_12312_12392	CTATGCCTTTTCTTGTTATGTTCATATATCAAATGATAATCAGAACTTAGAACAACTAGACAAGCTGTTAGACTCAAATT
NC_025350_Tuhoko_virus_3_M_12393_12473	GATATATCAACAGGTTATGATATTAGGACTTGGATTAATTGAAACTTGGCTGGCTGCGCCAAATACAGAAAATGAAGAAG
NC_025350_Tuhoko_virus_3_M_12474_12554	TATATCTGTACATCTGCACACAGGATCCTCATGTTGCATAAGACCTGTAGACATGTGTGTACTCAATGAAACTGAAGCTG
NC_025350_Tuhoko_virus_3_M_12555_12635	AGTCCCTCATCTACTCGTGCCGCGAGCAAACAAATTTGTATTTGATGACAATCCGTTGGATAATGAGCAAATTGAGATTA
NC_025350_Tuhoko_virus_3_M_12636_12716	AGACAATGTTATGATACAATCTAGACTGAGTGGAGTTGATTGCTTGACCATTCAAGAGAAGATACCTCTTCTCGCACATC
NC_025350_Tuhoko_virus_3_M_12717_12797	GATTGCCTTACAGTTTGCCCACAGCTTAATTGGCCTGGATGAATCTACCTCGTTAGTGAATGATGCAGTTGTGGATGCGG
NC_025350_Tuhoko_virus_3_M_12798_12878	TTATGCTGCAAATTGGATTAGTGAATGTTTGAATACTTATTTGGATAGGGTATTTTATTACACAGCATGGAACATCCTAC
NC_025350_Tuhoko_virus_3_M_12879_12959	TGACCTCTCTTACCAGATGTATTACATGAGGATTACCTCTGTTAATTCGGTGCTAGATTACCTAGGTATTGTCCTCGCAA
NC_025350_Tuhoko_virus_3_M_12960_13040	AATCCCAGGACTTGCTTTAAGTGGAATTGCCTCAACAATTAACCATCCTAAAATCCTAAGAAGAATGATAAATCTTGGAA
NC_025350_Tuhoko_virus_3_M_13041_13121	CTTGGTCCCATCTAACTCTCCTTACCTGGCAACACTCAATTATCACAAATTGACAACTGATGCAATTATGTGGGGTGCAC

NC_025350_Tuhoko_virus_3_M_13122_13202	TCAAGTGCTAGCTGATCTACAATCAGGAAGGGACATTGAAATCATAATACCTTCTGAAGATAGCACTGAACTGAGTGACA
NC_025350_Tuhoko_virus_3_M_13203_13283	AACTCTGAATTTGATAGCTAGAAAATTAACTCTTATGGCCCTTGTAGTTGCACCTGATTCAGCACTTCCTTATGTCAGGG
NC_025350_Tuhoko_virus_3_M_13284_13364	TCTTCCACCAGATCAGAAATGCAAGGTCTTAACAGATTATCTTGTACAGCTTGTGTTAACCATGACCTCAGATCCAGAAA
NC_025350_Tuhoko_virus_3_M_13365_13445	CATAAATAGCTGGAGATCAGTTATCCAAATGCCAAAGTTATCAGCATTTCCCCATAATCTATTCTACCTAACTAGGAAAA
NC_025350_Tuhoko_virus_3_M_13446_13526	GTTAAATGCTGTCAGGGACTCGTCTGAAGGACAAGCCTTACTGGAGCAGTTCTATGGGAGCTTCGGATTCTTAGAATCTG
NC_025350_Tuhoko_virus_3_M_13527_13607	TCTAAATGTCCAATTAATGGATCTTGCTTCTGGTTCTCAGGGTGATTCTAAAAGTCTAACAGTATTTGATGTAGTTCAAG
NC_025350_Tuhoko_virus_3_M_13608_13688	ATTGTTCAACTCGGCACATAATGTAGAACGATTGTGCTTTCCTACAGGAGACATCCAATTAGACCTAGAGCCGATAGGTC
NC_025350_Tuhoko_virus_3_M_13689_13769	TGTCACACCGCCAACTCATCATGTCCTAAGGCCAATTGGCCTCTCATCAACATCATGGTACAAGGGCATCACACTGATCA
NC_025350_Tuhoko_virus_3_M_13770_13850	TTATCTTAAAGGTCTTAAACTGCCCATAGGTGATCATCTTTATTTAGCTGAGGGAAGTGGGGCAATAATGACCATAATTG
NC_025350_Tuhoko_virus_3_M_13851_13931	AGCATACTTCCCTGGAGAGCACATATTTTACAATAGTTTTTTCACAAGTGGGCAATGCCCTCCACAGAGAAATTTCCAGC
NC_025350_Tuhoko_virus_3_M_13932_14012	AATGCCTACACAGTTTACTGAGAGCATAGTGTACCAGCATTTGCAAAATGACATACCCTGTGATATGGGATTTGTACAAG
NC_025350_Tuhoko_virus_3_M_14013_14093	CTTCATCCCATTGTGGAGTGGAAACAGTAAACAAACTGATTTGAGTCGTAAGGAGTGTGTCAATTTTATCCTAGATAGAA
NC_025350_Tuhoko_virus_3_M_14094_14174	ACCTTCTTCTACAGTCTCAATACTCTCCTGTGATTTAGAAGAGGGCTTTATAAGTAATCCAAACGAGTTATCTTCAGCCC
NC_025350_Tuhoko_virus_3_M_14175_14255	ACTTCATGTACTGTTATTATCATTCATTCTTGTCAAAGAGTCAGGTGTGACAATACTTAAAACTGACTTAATGCCGTTCT
NC_025350_Tuhoko_virus_3_M_14256_14336	AAAATTGTCAAAACTTGTTACAATACTAAGTCACAGATACACCAAGATTGATGCTGTCAGATCAGCCTACTCTGATCCTT
NC_025350_Tuhoko_virus_3_M_14337_14417	TCATCATGAAATCTTCTTAGTGTGTATTGCTGGTCCCGGGATGGGTATTGGGGAATTCAATATGGCTCTGCACAAAGCAA
NC_025350_Tuhoko_virus_3_M_14418_14498	TACTGATGCACAGCAAGGTTTCTCAATCATCAATCCGCTTCGTCATCATGAACTTCTTCATATGACTGCAGATTCGTCTA
NC_025350_Tuhoko_virus_3_M_14499_14579	TAACGTGATGGACATAATAGATATGCAAATCAAATTGGCTCAGGCGTCCGAGAATGAAGATGATCATATCCTGCTGTCTA
NC_025350_Tuhoko_virus_3_M_14580_14660	ACTGGGTTGCAGATCCCAGACAGATAAGCTACTGTCAATTAAAGCATCAAAGAGTATGGACGAGTTCCTTCATAGAGTGG
NC_025350_Tuhoko_virus_3_M_14661_14741	ATCACTTATCACTGTGTATCTAAAAGAATCCATCAACATCATTGAGTCTTACAATGAAGATCGGACTAGACTAATCTTTA
NC_025350_Tuhoko_virus_3_M_14742_14822	GGCCTATAATGTATCAGCTTATGGGAAAATTAAAACCTCAGCCAAAATATGTGCAAAGACAATTCTTGACATAACTATCC
NC_025350_Tuhoko_virus_3_M_14823_14903	AAATTGGACACTCCTCGGATTCCCTCTCAGAAAGCTGGTTGCTGCCAGTCTAAGGCTAGGAGAATTTTCAGCAGGCCTTG
NC_025350_Tuhoko_virus_3_M_14904_14984	AATTAATGCAAAAGATTTTCTTCAAAGATCTAATGCTAGGAAATATATTAAGACTTCAGTAGGATTAGAACAATTGGATA
NC_025350_Tuhoko_virus_3_M_14985_15065	TGAGCTATCCCAAAATTTGAGGCTATTGCTTAGCAGAGCTGAACAGAAGACTGTCTGGAAACAGATTGGAGCCATCAATC
NC_025350_Tuhoko_virus_3_M_15066_15146	GGTTAGTTCTCTCGAGAATGATTCAGATACTCTCTGGGGTGATGCATACCTAGAGGACGAACCAGAGCAACCTGAATTCG
NC_025350_Tuhoko_virus_3_M_15147_15227	TATCGCTGGCGAAGAAATTTAGAAAAGATGCAAGGTAAAATGCAAGCTAATGCAGTTTAAGAAAAAATTATTGATTTTCC
NC_025350_Tuhoko_virus_3_M_15155_15234	GGCGAAGAAATTTAGAAAAGATGCAAGGTAAAATGCAAGCTAATGCAGTTTAAGAAAAAATTATTGATTTTCCCCTTGGT
