# Supplementary material for: Sosuga Virus Detected in Egyptian Rousette Bats (Rousettus aegyptiacus) in Sierra Leone
Source: Viruses. 2024 Apr 22;16(4):648. doi: 10.3390/v16040648 (PMC11054331; doi:10.3390/v16040648)
Supplement: Supplementary file 1 [file viruses-16-00648-s001.zip › Table S2 Bat capture totals.pdf]

Table S2. Bats captured and tested for Sosuga virus (SOSV) in Sierra Leone. Of the 404 Egyptian rousette bats (ERB) captured, 27 were not sampled and tested for SOSV and therefore not used in the analyses of the 377 ERBs reported in the results. Only the ERBs had detectable SOSV RNA.

Species	Number captured
Eidolon helvum	101
Epomophorus gambianus	61
Epomops buettikoferi	481
Hipposideros abae	114
Hipposideros beatus	1
Hipposideros caffer	30
Hipposideros camerunensis	1
Hipposideros cyclops	7
Hipposideros fuliginosus	36
Hipposideros gigas	2
Hipposideros ruber	123
Hypsignathus monstrosus	114
Lissonycteris angolensis	5
Megaloglossus woermanni	16
Micropteropus pusillus	2
Mops condylurus	127
Myonycteris torquata	61
Nanonycetris veldkampii	13
Neoromicia somalica	1
Rhinolophus landeri	31
Rousettus aegyptiacus	404
Scotophilus nigrita	1
**Total**	**1732**
