# Supplementary material for: Sosuga Virus Detected in Egyptian Rousette Bats (Rousettus aegyptiacus) in Sierra Leone
Source: Viruses. 2024 Apr 22;16(4):648. doi: 10.3390/v16040648 (PMC11054331; doi:10.3390/v16040648)
Supplement: Supplementary file 1 [file viruses-16-00648-s001.zip › Table S3_GenBank.pdf]

Table S3. GenBank accession numbers for the Sosuga virus (SOSV) sequences and sequence fragments. Human and bat SOSV sequences are listed by species, location, unique field identification (ID), either whole genome (WG) or by hemagglutinin neuraminidase (HN) and nucleoprotein (NP) genes, and their GenBank accession numbers.

GenBank Accession Numbers					
Species	Location	Field ID	WG	HN	NP
Homo sapiens	South Sudan, Uganda, USA	---	NC025343	---	---
Rousettus aegyptiacus	Sierra Leone	Bat-1021	PP646291	---	---
	Uganda	Bat-926	---	---	KP150641
	Uganda	Bat-1271	---	KP150639	KP150651
	Uganda	Bat-1302	---	---	KP150642
	Uganda	Bat-1319	---	KP150638	KP150643
	Uganda	Bat-1392	---	---	KP150649
	Uganda	Bat-1516	---	---	KP150644
	Uganda	Bat-1541	---	---	KP150645
	Uganda	Bat-1571	---	---	KP150646
	Uganda	Bat-1605	---	KP150637	KP150647
	Uganda	Bat-1624	---	KP150640	KP150648
	Uganda	Bat-1450	---	---	KP150650

--- indicates no sequence.
